# Low‐density subculture: a technical note on the importance of avoiding cell‐to‐cell contact during mesenchymal stromal cell expansion

**DOI:** 10.1002/term.2051

**Published:** 2015-07-07

**Authors:** Richard Balint, Stephen M. Richardson, Sarah H. Cartmell

**Affiliations:** ^1^ School of Materials, Faculty of Engineering and Physical SciencesUniversity of ManchesterUK; ^2^ Centre for Tissue Injury and Repair, Institute of Inflammation and Repair, Faculty of Medical and Human SciencesUniversity of ManchesterUK

**Keywords:** mesenchymal stromal cells, culture, expansion, low confluence, proliferation

## Abstract

Numerous scientific studies and clinical trials are carried out each year exploring the use of mesenchymal stromal cells in regenerative medicine and tissue engineering. However, the effective and reliable expansion of this very important cell type remains a challenge. In this study the importance of cell‐to‐cell contact during expansion has been explored on the proliferation and differentiation potential of the produced cells. Cells were cultured up to passage 5 under conditions where cell‐to‐cell contact was either probable (40–70% confluence; see supporting information, Protocol A) or where it was unlikely (10–50% confluence; see supporting information, Protocol B). The effect of the two different conditions on expansion efficiency; proliferation rate and tri‐lineage differentiation potential was assessed. Differences in immunophenotype, cell size and senescence were also investigated. Protocol B cultures expanded twice as fast as those cultured with Protocol A. In passage 5 experiments low confluence expanded cells displayed a 10% higher overall proliferation rate, and produced 23% more cells in growth, 12% more in osteogenic, 77% more in adipogenic, but 27% less in chondrogenic medium. Differentiation potential wasn't decisively affected at the mRNA level. However, Protocol B favoured bone and cartilage differentiation at the secretional level. Protocol A populations showed reduced purity, expressing CD105 in only 76% compared to the 96.7% in Protocol B cultures. Protocol A populations also contained significantly more (+4.2%) senescent cells, however, no difference was found in cell size between the two protocols. The findings of this study suggest that cell‐to‐cell contact, and therefore high confluence levels, is detrimental to MSC quality. © 2015 The Authors Journal of Tissue Engineering and Regenerative Medicine Published by John Wiley & Sons Ltd.

1

Since their discovery over 40 years ago, mesenchymal stromal cells (MSCs) have become a widely used and invaluable tool for regenerative medicine (Chang *et al*., 2012; Colter *et al*., 2001; DiGirolano *et al*., 1999; Gregory *et al*., 2005; Sekiya *et al*., 2002; Smith *et al*., 2004). Today, numerous research studies and clinical trials are under way in an attempt to harness the therapeutic potential of this cell type (Jung *et al*., 2012). Their application requires the cells to be extensively expanded *ex vivo*, as donor tissue (bone marrow, adipose, etc.) contains only a small number of MSCs (Apel *et al*., 2009; Bartmann *et al*., 2007; Bonab *et al*., 2006; Jung *et al*., 2012; Lepperdinger *et al*., 2008). However, the effective and reliable expansion of this very important cell type remains a challenge. Currently, the generally applied culture techniques result in the cells progressively losing their self‐renewing and differentiation capability, from the first day in culture onwards. As a result of this, the cells cannot be effectively used beyond passages 4–5 (Banfi *et al*., 2000).

Furthermore, numerous studies have reported great variation between the behaviour of cells, not only from different donors but also from different cultures of the same donor. There appears to be no correlation between the variation in cell behaviour and the age, sex or race of the donor or the isolation source (Ankrum and Karp, 2010; Bernardo *et al*., 2007; Phinney *et al*., 1999; Siddappa *et al*., 2007). This also suggests that at least some of the donor variation witnessed in the literature stems from imperfect culture conditions, rather than from biologically inherent variation. Therefore, more and more researchers and clinicians across the field of regenerative medicine are emphasizing the importance of developing a robust and reliable standardized MSC culture procedure (Ankrum and Karp, 2010; Jung *et al*., 2012).

There is growing evidence in the literature that seeding MSCs at low densities (e.g. 10–1000 cells/cm^2^) (Sekiya *et al*., 2002; Sotiropoulou *et al*., 2006) down to clonal levels (0.5–6 cells/cm^2^) (Colter *et al*., 2000, 2001) produces cells with a higher proliferation rate and better maintains the tri‐lineage potential during expansion. We hypothesize that it is the lack of cell‐to‐cell contact in these low‐density cultures that provides these beneficial effects, and that the confluence at subculture is therefore the more important factor, rather than the density at plating.

In order to test this hypothesis, in this study MSCs were expanded using two different methods up to passage 5: one that enables cell‐to‐cell contact (culturing from 40% to 70% confluence; see supporting information, Protocol A) that is equivalent to the currently generally employed technique; and one in which cell‐to‐cell contact is unlikely (culturing from 10% to 50% confluence; see supporting information, Protocol B). The effect of the two protocols on the proliferation rate, immunophenotype, cell size, senescence and osteogenic, chondrogenic and adipogenic differentiation potential of the generated populations was assessed.

Commercial bone marrow‐derived human MSCs (Lonza, UK) were expanded from passage 2 using either Protocol A (40–70%) or Protocol B (10–50%) in growth medium (MSCGM Culture Medium, Lonza, UK) at 37 °C and 5% CO_2_ in a humidified atmosphere. These MSCs will be referred to as Protocol A and Protocol B cells. All experiments were carried out using passage 5 cells.

Osteogenic medium was prepared by supplementing base medium [low‐glucose Dulbecco's modified Eagle's medium (DMEM) containing l‐glutamine, 10% heat‐inactivated fetal bovine serum (FBS) and 1% antibiotics and antimycotics] with 10^–5^ mm dexamethasone, 10 mm *β*‐glycerolphosphate and 50 µg/ml ascorbic acid. Adipogenic differentiation medium was prepared by supplementing base medium with 0.5 µm dexamethasone, 0.5 mm isobutylmethylxanthine and 50 µm indomethacin. Chondrogenic medium was prepared by supplementing high‐glucose DMEM with 1% FBS, 10 ng/ml TGF‐*β*3, 10 µg/ml insulin, 5.5 µg/ml transferrin, 6.7 ng/ml selenium, 100 µm ascorbic acid‐2‐phosphate, 1.25 mg/ml bovine serum albumin (BSA), 10^−7^ m dexamethasone, 5.4 µg/ml linoleic acid, 40 µg/ml l‐proline and 100 U/ml penicillin, 100 µg/ml streptomycin, and 2.5 µg/ml amphotericin B.

The proliferation rates of Protocol A and B cells were examined from three perspectives. First, cell numbers were measured during expansions using C‐Chip haemocytometers upon plating and subculture of each passage. Data were collected from eight expansions carried out using Protocol A and 7 using Protocol B. Second, at passage 5, Protocol A and B cells were seeded at 1575 cells/cm^2^ culture density into 24‐well plates (3000 cells/well). The cells were cultured for up to 4 days in growth medium. Cell numbers (*n =* 6) were determined each day, using the PicoGreen dsDNA Assay Kit (Life Technologies). Third, the proliferations of Protocol A and B cells were investigated during tri‐lineage differentiation. For osteogenic and adipogenic differentiation, cells were plated into six‐well plates at 3150 cells/cm^2^ culture density and were cultured in growth, adipogenic and osteogenic media for 14 days, after which they were collected for PicoGreen assay (*n =* 4). Chondrogenic differentiation was performed by pellet culturing 250 000 cells for 21 days in chondrogenic medium; the cell pellets (*n =* 4) were harvested on day 21 for PicoGreen assay.

In order to assess the tri‐lineage potential of the generated MSCs, passage 5 cells were assayed for the gene expression (*n =* 4; Taqman technique, Life Technologies) of osteogenic (alkaline phosphatase, collagen type I, osterix, osteopontin, osteocalcin), chondrogenic (aggrecan, collagen type II) and adipogenic (adiponectin, leptin) markers after 14 days of differentiation in the appropriate medium formulations.

Alkaline phosphatase activity (*n =* 4) and lipid formation (oil red O, *n =* 4) were also measured after 14 days; while glycosaminoglycan content (DMMB assay, *n =* 4; Alcian blue, *n =* 2) was investigated after 21 days in the appropriate differentiated samples.

Phenotypic changes were detected through the flow cytometry of passage 5 populations, using the BD Stemflow hMSC Analysis kit (BD Biosciences), evaluating the expression of positive (CD105, CD73, CD90) and negative (CD45, CD34, CD11b, CD19, HLA‐DR PE) MSC markers.

In order to detect whether the loss of MSC quality is paralleled by a change in cell size, cells from both protocols were seeded into six‐well plates (*n =* 3) at a density of 5000 cells/cm^2^, and cultured for 24 h. The cells were stained for their membranes (CellMask Orange, Life Technologies) and nuclei (ProLong Gold with DAPI, Life Technologies) and imaged under a fluorescent microscope. Five images/sample were taken and analysed for cell size using CellProfiler software.

The percentage of senescent cells in passage 5 cultures was measured through *β*‐galactosidase (a marker of cellular senescence) staining (*n =* 4) (Sigma‐Aldrich).

Statistical analysis was performed using the commercial software GraphPad Prism v. 5.0 (GraphPad Software). The different treatments were compared using Student's *t*‐test, the Kolmogorov–Smirnov test and one‐ and two‐way ANOVA, depending on the experiment. Statistical differences with *p* ≤ 0.05 were considered significant.

Protocol B cells showed a significantly higher population doubling speed/day during expansion (*p =* 0.004), with Protocol B cultures requiring only 2.3 days on average to double their cell numbers, compared to the 6 days found with Protocol A cells (Figure 1A). Protocol B expansion was also more reliable, showing only ± 19% standard deviation (SD), in contrast with the ± 47% SD of Protocol A cultures. When maintained for 4 days in growth medium, passage 5 Protocol B cells showed significantly higher cell numbers from 48 h onward (*p <* 0.001) and a 10% higher overall proliferation rate than Protocol A cells (Figure 1B). Similarly, in various differentiation media, after 14 days of culture, Protocol B samples showed significantly higher cell numbers (*p <* 0.001), with 23% more cells being present in growth, 12% more in osteogenic and 77% more in adipogenic medium samples compared to Protocol A (Figure 1C). In chondrogenic pellet cultures, however, after 21 days Protocol A samples contained 27% more cells.

**Figure 1:**
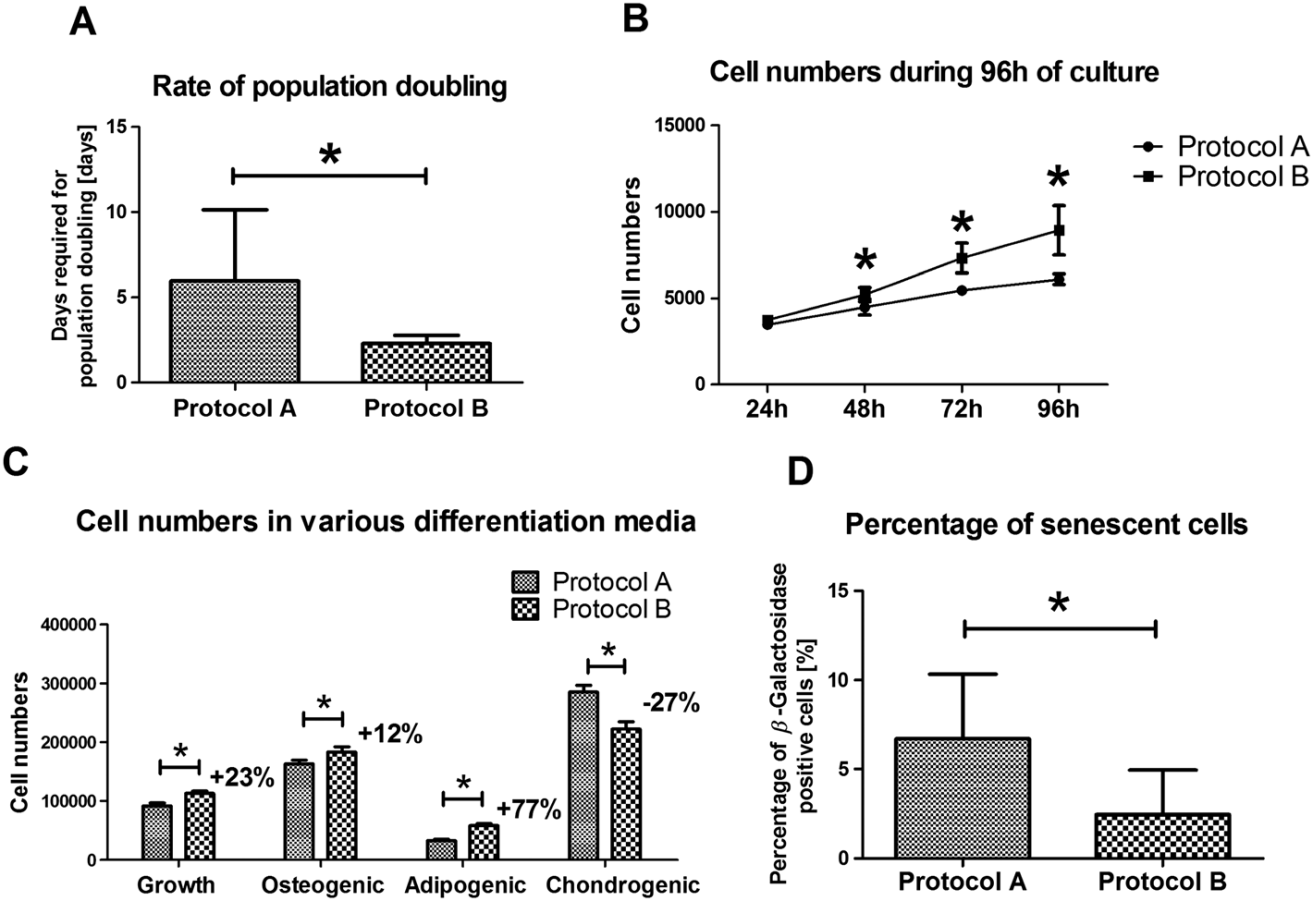
Proliferation of MSCs with the two protocols. (A) Rate of population doubling during expansion from passage 2 to passage 5. (B) Cell numbers of passage 5 Protocol A and B cells expanded for 96 h in growth medium. (C) Cell numbers in Protocol A and B samples in growth, osteogenic and adipogenic media after 14 days, and in chondrogenic pellet cultures after 21 days. (D) Percentage of senescent cells in Protocol A and B populations; *p < 0.05

Protocol B cultures showed high purity, displaying an expression profile of 96.7%, 100% and 97.3% positive for CD105, CD73 and CD90, respectively. In contrast, Protocol A cells showed reduced MSC characteristics, expressing CD105 in only 76% and CD90 in only 90.6% of the population.

Comparison of the cell sizes from the two regimes (1538 ± 837 µm^2^ for Protocol B and 1582 ± 1136 µm^2^ for Protocol A), using Student's *t*‐test, showed no significant difference. Furthermore, the Kolmogorov–Smirnov test showed that the size of the cells with the two treatments followed the same probability distribution function.

On the other hand, the results showed that a significantly higher (*p =* 0.0015) percentage of senescent (*β*‐galactosidase‐positive) cells were present in Protocol A populations (6.7%), compared to Protocol B (2.5%) (Figure 1D). Protocol B cells expressed significantly higher levels of osteopontin in osteogenic medium and osteocalcin in growth medium. Osteogenic chemical stimulation was able to enhance collagen type I expression in Protocol B, but not in Protocol A, cultures. However, all these fold increases were in the range of, or below, two‐fold. At the secretional level, Protocol B cells did show substantially higher alkaline phosphatase enzymatic activity (Figure 2A), suggesting that there was a difference between the osteogenic differential potential of the two cell cohorts.

**Figure 2:**
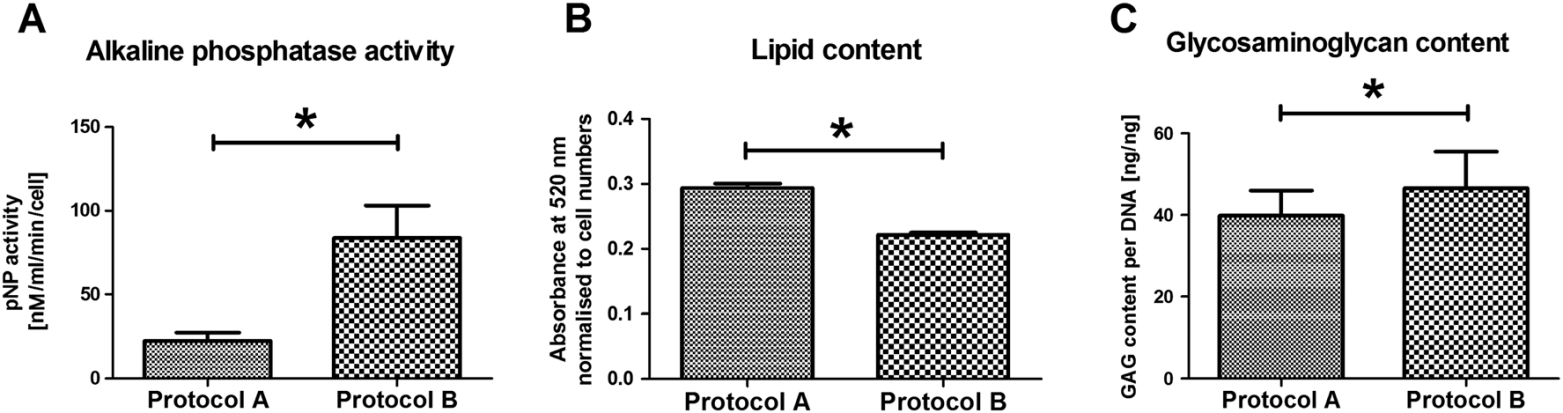
(A) Alkaline phosphatase activity, (B) lipid content and (C) glycosaminoglycan content in Protocol A and B cultures; *p < 0.05

Adiponectin mRNA expression was significantly higher (ca. three‐fold) with Protocol B cells in both growth and adipogenic medium. Protocol B samples displayed lower leptin levels in growth medium, while there was no statistically significant difference between the two protocols in adipogenic medium. Lipid formation per cell, measured colorimetrically through oil red O staining, was 32% higher (*p <* 0.001) in Protocol A samples (Figure 2B).

Collagen type 2 and aggrecan expression was comparable between the two protocols. Safranin O staining indicated low levels of glycosaminoglycan deposition with both protocols; quantitative analysis (DMMB assay), however, showed that Protocol B cells produced 16% more (*p =* 0.015) glycosaminoclycans (Figure 2C).

The results of this study demonstrate the importance of avoiding cell‐to‐cell contact in MSC cultures. High‐confluence cultures generated MSCs with a reduced proliferation rate, displaying lower cell numbers not just during expansion but in osteogenic and adipogenic differentiation experiments as well. This loss of self‐renewal capacity in high‐confluence cultures is paralleled by a loss of MSC phenotype and an increase in the number of senescent cells. Contrary to previous findings (Sekiya *et al*., 2002), however, cell size was not found to play an important role in this process.

Regarding the differentiation potential of the cells, no definitive difference was observed between the two culture methods at the gene expression level; however, at the secretional level, low‐confluence culture (Protocol B) favoured the osteogenic and chondrogenic pathways, while Protocol A appeared to promote adipogenic differentiation.

In conclusion, the findings of this study demonstrate that culture above 50% confluence impairs MSC self‐renewal and differentiation capacity, supporting the hypothesis that cell‐to‐cell contact is detrimental to MSC quality.

## Conflict of interest

The authors have declared that there is no conflict of interest.

## Supporting information

The following supporting information may be found in the online version of this article:

Supplementary methods: Protocol A

Supplementary methods: Protocol B

Expression of various osteogenic, chondrogenic and adipogenic marker genes, distribution of cell sizes and safranin O‐stained chondrogenic pellet cultures of differentiated Protocol A and B cultures
