# Supplementary material for: Low‐density subculture: a technical note on the importance of avoiding cell‐to‐cell contact during mesenchymal stromal cell expansion
Source: J Tissue Eng Regen Med. 2015 Jul 7;9(10):1200–3. doi: 10.1002/term.2051 (PMC4858810; doi:10.1002/term.2051)
Supplement: Supplementary file 1 — Supplementary methods: Protocol A [file TERM-9-1200-s001.doc]

**Balint *et al* – Supplementary Information 1**

**PROTOCOL A – The “traditional” protocol that follows the supplier’s recommendations**

**Initiating the cell culture:**

- Cells are plated at the recommended density of **5,000-6,000 cells per cm^2^**. For a T75 flask this is 375.000-450.000 cells per flask.

**Maintenance of the culture:**

- The cell culture is inspected 24h after initiation in order to assess cell viability. The culture medium is changed post-assessment to remove any dead cells.
- Cells are inspected and the culture medium is changed every 3 or 4 days.

**Sub-culturing the cells:**

- Manufacturers highlight the importance of contact inhibition and recommend sub-culturing at 90% confluence. However, in this study a cell density –**70% confluence** – smaller than the recommended confluence was chosen as the limit.
- Judged by optical microscopy, if the cells are deemed approx. 70% confluent cells are harvested for further expansion, freezing down or experiments.
- If the cells are not deemed to be at 70% confluence they are kept in culture until they achieve this cell density.
- Cells are counted at the initiation and at the harvesting of each passage in order to enable the accurate tracking of the performance of the culture.

Generally speaking commercial hMSCs are delivered at passage 2. In this study, cells were expanded up to passage 4 and were used at passage 5 to ensure that their multi-potency has not been compromised due to prolonged monolayer culturing.
