# Supplementary material for: Low‐density subculture: a technical note on the importance of avoiding cell‐to‐cell contact during mesenchymal stromal cell expansion
Source: J Tissue Eng Regen Med. 2015 Jul 7;9(10):1200–3. doi: 10.1002/term.2051 (PMC4858810; doi:10.1002/term.2051)
Supplement: Supplementary file 2 — Supplementary methods: Protocol B [file TERM-9-1200-s002.doc]

**Balint *et al* – Supplementary Information 2**

**PROTOCOL B – Low confluence expansion protocol**

**Initiating the cell culture:**

- hMSCs are plated at a low density of **1000-1350 cells per cm^2^**. For a T75 flask this is 75.000-100.000 cells per flask.

**Maintenance of the culture:**

- The cell culture is inspected 24h after initiation in order to assess cell viability. The culture medium is changed post-assessment to remove any dead cells.
- Cell cultures are inspected daily to assess the confluence of the culture.

**Sub-culturing:**

- After 4 days hMSCs cultures reach approx. **50% confluence** and are sub-cultured, frozen down or used for experiments.
- Very importantly, cells are not allowed to expand above 50% confluence, as permitting the cells to do so may result in a decreased proliferation rate, altered morphology and a loss in differentiation potential.
- **Culture time is kept consistent** throughout all expansions and passages:
  I.e. cell are always kept in culture for 4 days and then sub-cultured or used for experiments. (It is also desirable to keep the culture time consistent between different donors, though this might not be possible due to variations in proliferation rate.)
- Cells are counted at the initiation and at the harvesting of each passage in order to enable the accurate tracking of the performance of the culture.

Generally speaking commercial hMSCs are delivered at passage 2. In this study, cells were expanded up to passage 4 and were used at passage 5 to ensure that their multi-potency has not been compromised.
