# Supplementary figures and images for: Low‐density subculture: a technical note on the importance of avoiding cell‐to‐cell contact during mesenchymal stromal cell expansion
Source: J Tissue Eng Regen Med. 2015 Jul 7;9(10):1200–3. doi: 10.1002/term.2051 (PMC4858810; doi:10.1002/term.2051)

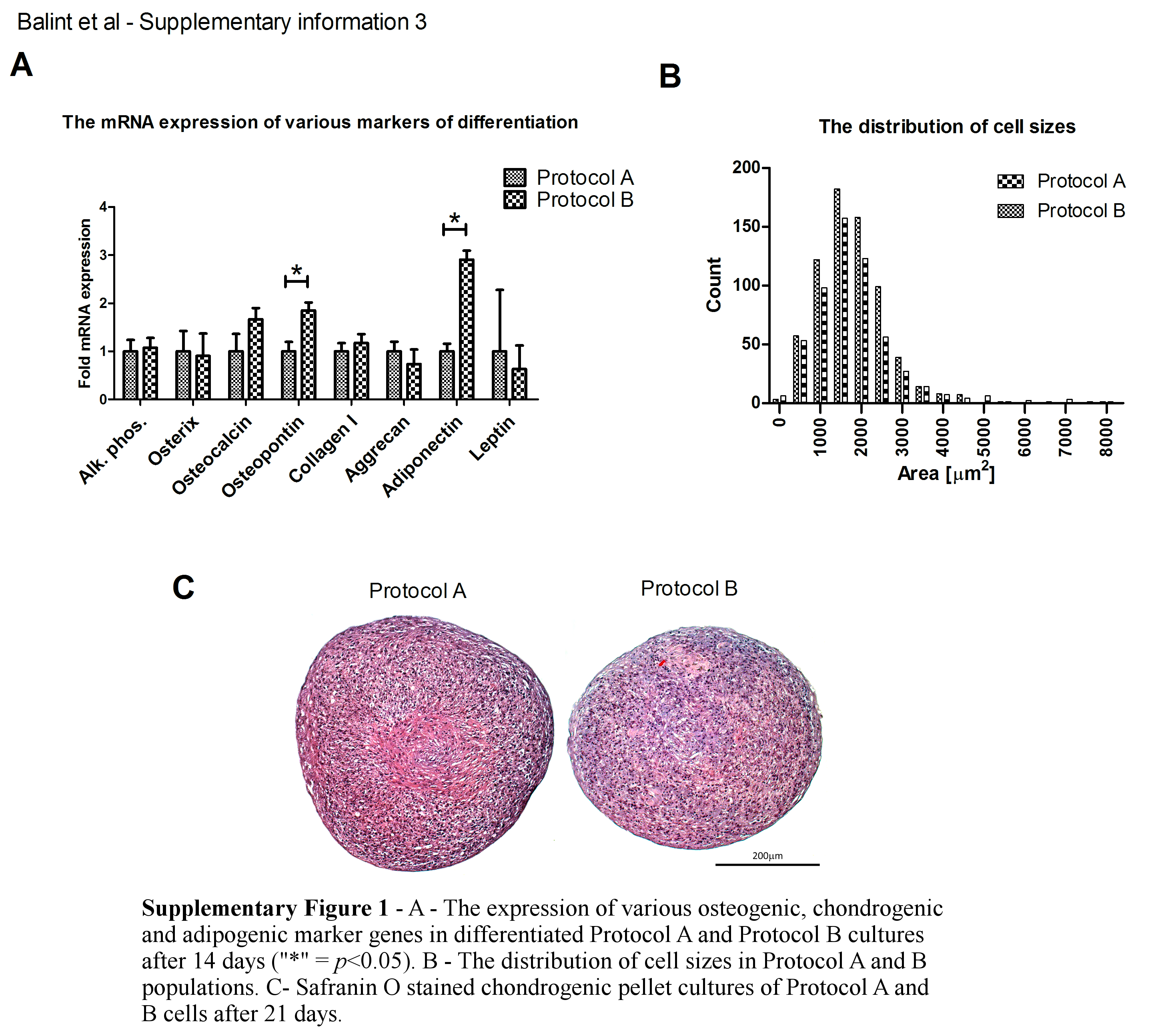

Supplement: Supplementary file 3 — Expression of various osteogenic, chondrogenic and adipogenic marker genes, distribution of cell sizes and safranin O‐stained chondrogenic pellet cultures of differentiated Protocol A and B cultures [file TERM-9-1200-s003.tif]
